# Interobserver and Intraobserver Reproducibility with Volume Dynamic Contrast Enhanced Computed Tomography (DCE-CT) in Gastroesophageal Junction Cancer

**DOI:** 10.3390/diagnostics6010008

**Published:** 2016-02-01

**Authors:** Martin Lundsgaard Hansen, Eva Fallentin, Thomas Axelsen, Carsten Lauridsen, Rikke Norling, Lars Bo Svendsen, Michael Bachmann Nielsen

**Affiliations:** 1 Department of Radiology, University Hospital of Copenhagen, Rigshospitalet, DK-2100 Copenhagen, Denmark; eva.fallentin@regionh.dk (E.F.); thomas.axelsen@regionh.dk (T.A.); carsten.lauridsen@regionh.dk (C.L.); rna@dadlnet.dk (R.N.); mbn@dadlnet.dk (M.B.N.); 2 Department of Radiology, Koege and Roskilde Hospital, DK-4000 Roskilde, Denmark; 3 Metropolitan University College, Radiography Education, Copenhagen, DK-2200 Copenhagen, Denmark; 4 Department of Surgery, University Hospital of Copenhagen, Rigshospitalet, DK-2100 Copenhagen, Denmark; lars.bo.svendsen@regionh.dk

**Keywords:** dynamic contrast enhanced computed tomography, imaging biomarkers, gastroesophageal cancer, CT perfusion, reproducibility

## Abstract

The purpose of this study was to assess inter- and intra-observer reproducibility of three different analytic methods to evaluate quantitative dynamic contrast-enhanced computed tomography (DCE-CT) measures from gastroesophageal junctional cancer. Twenty-five DCE-CT studies with gastroesophageal junction cancer were selected from a previous longitudinal study. Three radiologists independently reviewed all scans, and one repeated the analysis eight months later for intraobserver analysis. Review of the scans consisted of three analysis methods: (I) Four, fixed small sized regions of interest (2-dimensional (2D) fixed ROIs) placed in the tumor periphery, (II) 2-dimensional regions of interest (2D-ROI) along the tumor border in the tumor center, and (III) 3-dimensional volumes of interest (3D-VOI) containing the entire tumor volume. Arterial flow, blood volume and permeability (*k*^trans^) were recorded for each observation. Inter- and intra-observer variability were assessed by Intraclass Correlation Coefficient (ICC) and Bland-Altman statistics. Interobserver ICC was excellent for arterial flow (0.88), for blood volume (0.89) and for permeability (0.91) with 3D-VOI analysis. The 95% limits of agreement were narrower for 3D analysis compared to 2D analysis. Three-dimensional volume DCE-CT analysis of gastroesophageal junction cancer provides higher inter- and intra-observer reproducibility with narrower limits of agreement between readers compared to 2D analysis.

## 1. Introduction

Dynamic contrast-enhanced computed tomography (DCE-CT) is a functional imaging technique to measure tumor vascularization *in vivo* [1]. Tumor angiogenesis is essential in tumor growth, and DCE-CT could serve as a functional diagnostic tool to supplement morphologic imaging in terms of tumor characterization and response evaluation [2,3,4,5,6].

DCE-CT analysis is based on serial image acquisitions during contrast administration, which by different kinetic models can estimate the exchange of contrast between the intravascular space and the interstitial space in terms of blood flow, blood volume and permeability. Dedicated software generates quantitative maps with perfusion parameters after user definition of the arterial input [7]. Implementation of a DCE-CT scan protocol is relatively straight forward, and the wide availability of scanners compared to other functional imaging modalities makes it an interesting approach in medical imaging. Multidetector CT scanners can cover up to 16 cm in the *z*-axis with fixed table position, thereby enabling perfusion studies of entire organs or tumors without loss of temporal uniformity [8].

Reproducibility has previously been assessed in DCE-CT [9,10,11,12] with varying results, which could be attributed to tumor heterogeneity. Volume perfusion enables whole tumor coverage, and could thereby limit the variability caused by heterogeneity.

The aim of this study was to assess the inter- and intra-observer agreement in the reading of perfusion studies of gastroesophageal junction cancer with three methods, (I) Average perfusion measures derived from four separate, fixed sized regions of interest in the tumor periphery (2-dimensional (2D) fixed ROIs), (II) 2-dimensional free-hand drawn region of interest (2D-ROI) along the tumor border in the tumor center, and (III) 3-dimensional volume of interest (3D-VOI) covering the entire tumor volume. Our hypothesis was that 3D-VOI improved the inter- and intra-observer agreement on reading blood flow, blood volume and permeability compared to 2D measures.

## 2. Experimental Section

### 2.1. Patients

Twenty-five consecutive abdominal DCE-CT scans of gastroesophageal junction cancer were selected from a previous longitudinal study investigating perfusion changes in gastroesophageal junction cancer and gastric cancer during pre-operative chemotherapy [13]. The original study population also included five patients with primary gastric cancer and these cases were excluded from this study because they were anatomically different and hence would not fit into the three analysis methods described below. The study population consisted of 23 males and two females, mean age 65. All patients had biopsy confirmed adenocarcinoma of Gastro-Esophageal Junction (GEJ), and were eligible for pre-operative chemotherapy and surgery. All scans for this study were performed prior to chemotherapy. The research protocol was approved by the Committees on Biomedical Research for the Capital Region of Denmark (protocol number H-1-2010-132). All patients gave oral and written informed consent according to the Helsinki II Declaration.

### 2.2. Dynamic Contrast Enhanced CT Analysis

The scanning parameters, contrast media and patient preparation details are listed in List 1. Three radiologists independently analyzed each perfusion scan as described in the following section. Reader 1 (Eva Fallentin) was a Consultant Radiologist with 20 years of experience in abdominal radiology, and two years of experience in DCE-CT. Reader 2 (Thomas Axelsen) was a Consultant Radiologist with seven years of experience in abdominal radiology and one year of experience in DCE-CT. Reader 3 (Martin Lundsgaard Hansen) was a Resident Radiologist with two years of experience in DCE-CT. Readers 1 and 2 assessed inter-observer reproducibility, and Reader 3 assessed intraobserver reproducibility.

**Figure 1:**
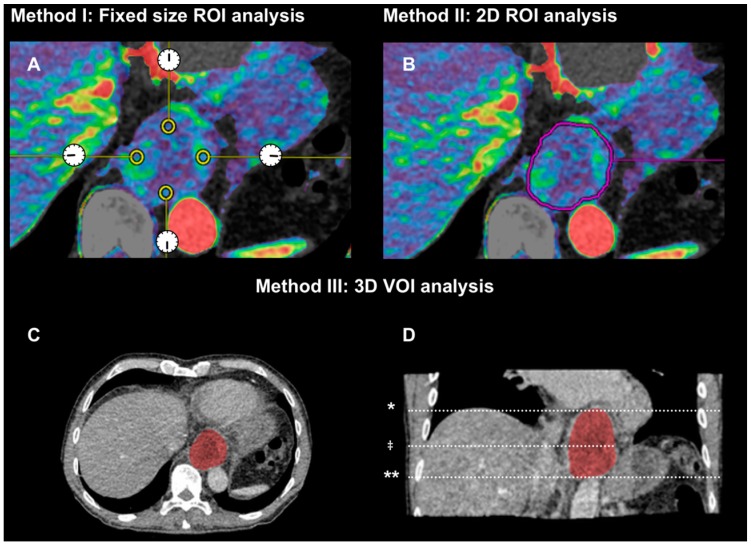
Illustration of three analysis methods. (**A**) method I includes four circular ROIs of fixed size (25–30 mm^2^) at 12, 3, 6 and 9 o’clock in tumor periphery at the tumors center in the *z*-axis; (**B**) method II is a 2D free-hand ROI along tumor border in the center slice; (**C** + **D**) method III is a 3D volume of interest covering the entire tumor volume. (**C**) illustrates a single axial image and (**D**) illustrates the tumor volume in a reconstructed coronal plane. (*) marks tumor start level, (**) marks tumor end level, and (^‡^) marks tumor center level for methods I and II. The illustrated case had a tumor volume of 194 mL.

For data analysis, the input artery was selected by placing a 100 mm^2^ circular ROI in the center of the abdominal aorta and a second ROI was placed in the tumor. Parametric perfusion maps were generated for Arterial flow (maximum slope method, single compartment), blood volume and permeability (*k*^trans^) (Patlak method, double compartment). Each perfusion scan was analyzed with three different approaches by the two readers. Reader 3 re-evaluated the perfusion scans ten months later for intra-observer agreement. The different analysis methods are also illustrated in Figure 1.

List 1 **Scan parameters, contrast media administration and patient preparation.**

Scan protocol and parameters

- 320-detector row CT scanner (Aquilion ONE, Toshiba Medical Systems, Ohtawara, Japan)
- *z*-axis coverage 12–16 cm
- 100 kV and 100 mA
- 0.5 s/rotation time and a fixed table position
- 19 consecutive scan volumes with variable start delay of 7.5 to 13.5 s determined by a test-bolus. Scan duration 55 to 60 s
- Iterative reconstruction (Adaptive Iterative Dose Reduction AIDR 3D (strong level) (Toshiba Medical Systems)
- Non-rigid 3D motion correction
- Image analysis on a stand-alone workstation (Vitrea 6.3, Vital Images, Toshiba Medical Systems, Minnetonka, MN, USA) for DCE-CT analysis

Contrast media

- Omnipaque 350; GE Healthcare, Milwaukee, WI, USA
- 30 to 40 mL, depending on bodyweight (<50 kg: 30 mL, 50–79 kg: 35 mL, >80 kg: 40 mL)
- Injection rate 5 to 8 mL/s (overall contrast injection time did not for any patient exceed 5 s)
- Saline flush of 30 mL

Patient preparation

- Two hours fast prior to the examination
- 500 mL of water per oral prior to the examination
- 20 mg hyoscine butylbromide (Buscopan, Boehringer Ingelheim, Ingelheim, Germany) intravenously
- Shallow free breathing with an abdominal strap

**Figure 2:**
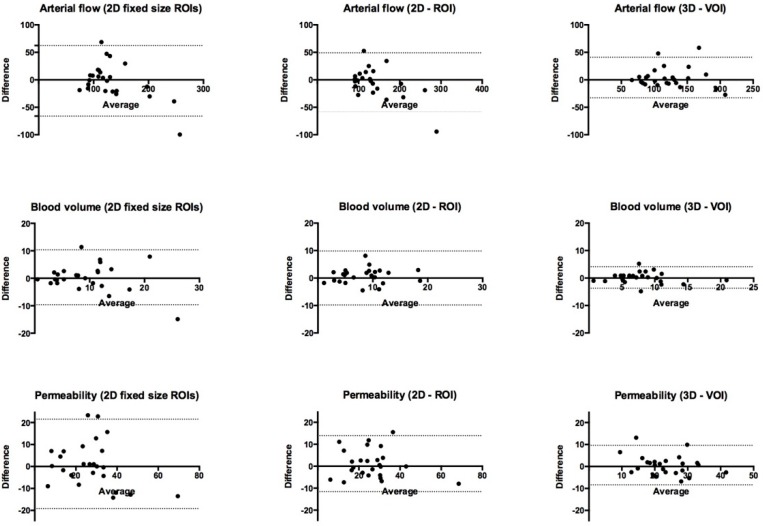
Bland–Altman plots for interobserver variation for arterial flow, blood volume and permeability with the three methods: (I) average between four ROIs of fixed size, (II) 2D-ROI around tumor border, and (III) 3D-VOI of the entire tumor volume. Narrowing of 95% limits of agreement for all three perfusion measures indicate better reproducibility for 3D-VOI compared to 2D analysis.

#### 2.2.1. Method (I): Small Region of Interests of Fixed Size in Tumor Periphery (2D Fixed ROIs)

The center level of the tumor was selected on a conventional CT scan with the largest cross-sectional area as the most representative level of the tumor. Small ROIs were placed (25–30 mm^2^) in the tumor periphery at 12, 3, 6, and 9 o’clock. The image number (tumor level) and perfusion parameters from each ROI were recorded. Average arterial flow, blood volume and permeability were calculated from the four ROIs.

#### 2.2.2. Method (II): 2D Region of Interest (2D-ROI) around Tumor Border at the Center Level of Tumor

A representative level of tumor was selected and a free-hand ROI was drawn around the tumor border, excluding the lumen and extratumoral tissue. The image number (tumor level) was noted, area of the ROI and perfusion parameters (arterial flow, blood volume and permeability) were measured.

#### 2.2.3. Method (III): 3D Volume of Interest (3D-VOI) Encompassing Entire Tumor Volume

By using a sculpt tool, a volume covering several images was defined as the tumor volume. Lumens and extratumoral tissues were excluded as mentioned in the two methods above. The first and last image number were noted, tumor volume and perfusion parameters (arterial flow, blood volume and permeability) were measured. List 2 summarizes the data recorded from the study.

List 2 **Measures recorded from Reader 1 and Reader 2 during readings with methods I, II and III.**

I: 2D fixed ROIs (Average between four ROIs placed in tumor periphery)

- Image number
- Average values for arterial flow (mL·min^−1^·100 g^−1^), blood volume (mL·100 g^−1^) and permeability (mL·min^−1^·100 g^−1^) from four ROIs at 12, 3, 6, and 9 o’clock

II: 2D-ROI (Region of interest along tumor border)

- Image number
- Tumor area (mm^2^)
- Average values for arterial flow, blood volume and permeability

III: 3D-VOI (Volume of interest covering the entire tumor)

- Start level (image number from top)
- End level (image number from top)
- Tumor length (mm)
- Tumor volume (mL)
- Average values for arterial flow, blood volume and permeability

### 2.3. Statistics

Perfusion variables derived from the analysis were evaluated for normal distribution using the Kolmogorow–Smirnow test. A paired *t*-test was used to compare tumor length and tumor volume between the readers. Intra- and interobserver reliability was calculated using Bland–Altman statistics with 95% limits of agreement [14] in Prism (Version 6, GraphPad Software, La Jolla, CA, USA). Correlation between observations were calculated using a two-way random, single measure (absolute agreement) Intraclass Correlation Coefficients (ICCs, model 2,1) for analysis method (I), (II) and (III) using SPSS for mac (version 20, IBM, Armonk, NY, USA). An ICC value below 0.40 was considered poor reliability, fair for values between 0.41 and 0.59, good for values between 0.60 and 0.74, and excellent for values between 0.75 and 1.00 (15). *p* values below 0.05 were considered statistically significant.

## 3. Results and Discussion

A total of 100 DCE-CT readings (25 readings from Reader 1, 25 readings from Reader 2 and 50 readings (2 × 25) from Reader 3) were available for analysis. Average reading time for Reader 3 was 11 min, which included loading, setting parameters for analysis and drawing ROIs according to the three described methods.

### 3.1. Bland–Altman Limits of Agreement and Intraclass Correlation Coefficient (ICC)

Table 1 and Table 2 summarize findings of Bland-Altman limits of agreement and ICC for arterial flow, blood volume and permeability measured for the three methods of analysis: (I) 2D fixed ROIs, (II) 2D-ROI and (III) 3D-VOI. Interobserver ICC was excellent for arterial flow (0.93), blood volume (0.94) and permeability (0.86) with 3D-VOI analysis. For 2D analysis (methods I and II), ICC was fair to good (0.49–0.71) for all perfusion parameters. Intra-observer ICC (Reader 1) for 3D-VOI was excellent for arterial flow (0.96) and blood volume (0.83). Intra-observer ICC (Reader 3) for 3D-VOI permeability was good (0.60). The span of limits of agreement was narrower for all parameters in inter- and intra-observer with 3D-VOI analysis (III) compared to 2D analysis. Inter-observer comparison between the specialists (Readers 1 and 2) and the resident (Reader 3) showed similar trends with narrower limits of agreement with 3D-VOI analysis (data not shown).

**Table 1:** Inter-observer reliability between readers.

CT Perfusion Parameter and Method	Bland–Altman 95% Limits of Agreement	Interobserver ICC
*Arterial flow mL·min^−1^·100 g^−1^*		
(I) 2D fixed ROIs	128.4 (−66.1; 62.3)	0.79 (0.57–0.90)
(II) 2D-ROI	107.5 (−58.4; 49.1)	0.88 (0.74–0.94)
(III) 3D-VOI	73.8 (−32.8; 41.0)	0.88 (0.75–0.95)
*Blood volume mL·100 g^−1^*		
(I) 2D fixed ROIs	20.0 (−9.6; 10.4)	0.70 (0.42–0.86)
(II) 2D-ROI	19.6 (−9.8; 9.8)	0.70 (0.42–0.86)
(III) 3D-VOI	7.8 (−3.7; 4.1)	0.89 (0.77–0.95)
*Permeability (k^trans^) mL·min^−1^·100 g^−1^*		
(I) 2D fixed ROIs	40.6 (−19.1; 21.5)	0.76 (0.52–0.88)
(II) 2D-ROI	25.6 (−11.6; 14.0)	0.87 (0.73–0.94)
(III) 3D-VOI	18.0 (−8.4; 9.6)	0.91 (0.90–0.96)

For Intraclass Correlation Coefficient (ICC) interobserver reliability tests, the first reading from reader 1 was selected. Span of limits of agreement and 95% limits of agreement derived from Bland–Altman test.

**Table 2:** Intra-observer reliability between readings. Span of limits of agreement and 95% limits of agreement derived from Bland–Altman test.

CT Perfusion Parameter and Method	95% Limits of Agreement	Intraobserver ICC
*Arterial flow mL·min^−1^·100 g^−1^*		
**(**I) 2D fixed ROIs	176.1 (−92.5; 83.6)	0.70 (0.42–0.85)
(II) 2D-ROI	159.0 (−82.6; 76.4)	0.72 (0.45–0.86)
(III) 3D-VOI	76.6 (−37.7; 38.9)	0.88 (0.75–0.95)
*Blood volume mL·100 g^−1^*		
(I) 2D fixed ROIs	15.9 (−7.8; 8.1)	0.77 (0.53–0.89)
(II) 2D-ROI	12.3 (−5.8; 6.5)	0.83 (0.65–0.92)
(III) 3D-VOI	8.0 (−3.8; 4.2)	0.89 (0.76–0.95)
*Permeability (k^trans^) mL·min^−1^·100 g^−1^*		
(I) 2D fixed ROIs	46.1 (−25.6; 20.5)	0.76 (0.53–0.89)
(II) 2D-ROI	43.6 (−24.0; 19.6)	0.72 (0.46–0.87)
(III) 3D-VOI	30.8 (−17.4; 13.4)	0.80 (0.55–0.91)

### 3.2. Tumor Definition and Delineation

There was no significant difference in selecting tumor center (*p* = 0.2), area of center tumor (*p* = 0.07) and tumor volume (*p* = 0.5) between readers 1 and 2.

### 3.3. Discussion

Reproducible data are essential for a new imaging modality to achieve acceptance in a clinical setting. The aim of this study was to examine reproducibility in reading DCE-CT studies of gastroesophageal cancer with three different approaches, and we showed that the limits of agreement were narrower for volumetric analysis compared to 2D ROI analysis, although all the methods had relatively wide limits of agreement in both inter- and intra-observer reproducibility. To our knowledge, this is the first reproducibility study on volumetric DCE-CT in gastroeosphageal cancer.

Physiological measures derived from DCE-CT analysis are the sum of true tissue perfusion, physiological variation, methodological variability in image acquisition and the variability introduced at the level of analysis [12]. This study addresses the variability introduced by the reader during analysis at the workstation. Our data shows no statistical difference in ICC between 2D and 3D analysis but narrower limits of agreement in 3D analysis. Interestingly, there was higher agreement between the two specialists (interobserver) compared to the residents two readings (intraobserver). One explanation could be higher routine and better agreement between specialists as reading and interpretation of gastro esophageal cancer on CT scans is difficult and generally regarded a specialist task. Ng *et al.* [11] showed that for lung tumors, the reproducibility increased with larger *z*-axis coverage (10 to 40 mm), thereby enabling the ability to measure the whole-tumor perfusion values. Chalian *et al.* [15] examined the interobserver variability between ROI analysis and VOI analysis of hepatic metastasis of colorectal cancer, and found a similar narrower limit of agreement in favor of volume analysis. On the other hand, Goh *et al.* [10] examined 10 patients with colorectal cancers and found the same variability with varying coverage from 5 to 20 mm. Intratumoral heterogeneity can cause a higher variability in measurements in a single plane. Volumetric analysis on the other hand enables the reader to better exclude non-viable tissue or large vascular structures. It has been shown that automated software for setting parameters for the kinetic models could result in better agreement between readers [16], and these results may point out the direction for future analysis software.

The relatively large limits of agreement demonstrated in our study challenges the use of DCE-CT in a clinical setting. DCE-CT has been applied in studies to measure therapy-induced changes in the tumors’ vascularity. For hepatocellular carcinomas [17], it has been shown that decreases of more than 35% in blood flow, 43% in blood volume and 93% in permeability were beyond variability in analysis, and could therefore serve as limits for response assessment. The limits of agreement in our interobserver analysis correspond to a decrease of 20% in arterial flow, 39% in blood volume and 36% in permeability. In clinical practice, biological variation in repeated scans will also play a role, making even larger limits necessary. Harders *et al.* [18] examined 59 patients with a single DCE-CT suspected lung malignancy and found out that DCE-CT could not discriminate between benign and malignant lesions partly because of the wide limits of agreement. 3D analysis is time consuming compared to 2D analysis and is typically done on a dedicated workstation, which does not favor its use in a clinical setting.

Research within the field of DCE-CT has been challenged by the lack of standards making inter-study comparison difficult. In 2012, Miles *et al.* published the Experimental Cancer Medicine Centre (ECMC) Network consensus document [19], and proposed recommendations for both scan protocols and for reporting data derived from DCE-CT. The required scan length is primarily dependent on the selected analysis model, and Miles *et al.* [19] recommends a total scan duration of 60–75 s when using Patlak analysis. Our protocol with a scan duration of 55–60 s was just short of this recommendation. Another fundamental requirement for DCE-CT analysis is maintaining temporal alignment of the targeted tissue for correct perfusion calculations. Tumors of the gastrointestinal tract are affected by motion from both peristalsis and respiratory movement of the diaphragm and anterior abdominal wall. Without taking measures to reduce and compensate for motion, the tumor borders can become fuzzy, impairing perfusion parameters [7]. It has been shown that a free breathing protocol requires less post-processing compared to a breath-hold scan protocol [20]. We placed an abdominal strap around the patient to hinder movement artefacts from anterior wall movement [21,22], and it was our experience that this measure also helped reminding the patient to breath shallow. We used hyoscine butylbromide as anti-peristaltic drugs which is generally recommended in DCE-CT imaging of the bowel or pelvis to hinder movement [2,7,10]. Lastly, we applied an advanced semi automated 3-dimensional non-rigid motion correction model, which is recommended in volumetric DCE-CT [19]. Motion correction software is typically vendor specific and is implemented in various forms making universal recommendations troublesome.

Many tumors exhibit heterogeneous perfusion [23]. We observed a substantial difference between each of the four ROIs used in method I, demonstrating this intratumoral heterogeneity. By averaging these four ROIs, the limits of agreement came close to the results derived from 2D-ROI analysis. Furthermore, 3D-VOI analysis (method III) averages the intratumoral heterogeneity in all planes and resulted in higher ICC and narrower limits of agreement, suggesting that volumetric perfusion analysis is more suitable for clinical use. On the other hand, average measurements does not reflect the distribution of perfusion inside the tumor volume [24]. Tumors respond differently to treatment with chemotherapy, radiotherapy, embolization or cryoablation, and, in some instances, it could be more attractive to report residual vascular “hot spots” [25] instead of perfusion averages. Analytic methods such as histogram analysis [26] or texture analysis [27] could also be a future direction for optimizing the reading of perfusion analysis.

Our study only demonstrates the variability in data analysis, and does not address the importance of repeatability in DCE-CT. The variability in data analysis was caused both by the setting of analysis parameters and the actual delineation of the tumor. The variability of setting the analysis parameters could have been further investigated by letting the two readers analyze the data with the same initial setting of arterial input and Patlak phase.

## 4. Conclusions

In conclusion, 3D volumetric analysis of DCE-CT studies has narrower limits of agreement in gastroesophageal junction cancer compared to 2D analysis. Tumors at the gastroesophageal junction are difficult to delineate, and there is a difference between readers in defining the cranial and caudal border of the tumor, although this did not affect the final tumor volume. Future studies are needed to address whether average tumor perfusion measures and more complex analytic methods such as texture analysis are suited for reporting clinical relevant values from DCE-CT studies.
